# Liquid Viscosity Sensor Using a Surface Acoustic Wave Device for Medical Applications Including Blood and Plasma

**DOI:** 10.3390/s23135911

**Published:** 2023-06-26

**Authors:** Kun-Lin Lee, Glen Kowach, Fang Li, Ioana Voiculescu

**Affiliations:** 1 Department of Mechanical Engineering, The City College of the City University of New York, New York, NY 10031, USA; voicules@ccny.cuny.edu; 2 Department of Chemistry and Biochemistry, The City College of the City University of New York, New York, NY 10031, USA; gkowach@ccny.cuny.edu; 3 Department of Mechanical Engineering, New York Institute of Technology, Old Westbury, NY 11568, USA; fli08@nyit.edu

**Keywords:** surface acoustic waves, SH-SAW, quartz, ZnO, waveguide, piezoelectric thin-film

## Abstract

Blood viscosity is the defining health indicator for hyperviscosity syndrome patients. This paper introduces an alternative approach for the real-time monitoring of blood viscosity by employing a surface-horizontal surface acoustic wave (SH-SAW) device at room temperature. A novel bi-layer waveguide is constructed on top of the SAW device. This device enables the SAW sensing of liquid droplets utilizing a bi-layer waveguide, consisting of a zinc oxide (ZnO) enhancement layer and Parlyene C, that facilitates the promotion of the surface horizontal mode. The ZnO piezoelectric thin-film layer enhanced the local particle displacement and dielectric coupling while the Parylene C layer constrained the wave mode at the interface of the piezoelectric material and polymer material. The device was tested with a liquid drop on the SAW delay-line path. Both experimental and finite element analysis results demonstrated the benefits of the bi-layer waveguide. The simulation results confirmed that the displacement field of local particles increased 9 times from 1.261 nm to 11.353 nm with the Parylene C/ZnO bi-layer waveguide structure. The device demonstrated a sensitivity of 3.57 ± 0.3125 kHz shift per centipoise enabling the potential for high precision blood viscosity monitoring.

## 1. Introduction

Blood viscosity depends on several factors such as hematocrit, plasma factors, red blood cell (RBC) deformability, and RBC aggregation [1]. An individual may be affected by one or more dysfunctional factors that might lead to impaired blood flow, which directly impacts drug delivery, oxygen exchange, and more. Hyperviscosity syndrome [2] is indicative of increased elements in the serum, reduced blood flow, or reduced RBC deformability. When vascular function cannot respond normally to the change in blood viscosity, the increased resistance may cause vaso-occlusive crises. Increased blood viscosity may cause thromboembolic events which is a concern especially among smokers. Research has shown the effect on hematological parameters, which relate the changes in blood viscosity, from tobacco users [3]. A single-use (disposable) device capable of blood viscosity detection is needed for health monitoring.

Surface acoustic wave (SAW) devices have been proposed for Internet of Things (IOT) applications, such as strain sensors [4,5], temperature sensors [6], and humidity sensors [7]. A wireless Rayleigh-type SAW device was designed for blood pressure detection [8]; however, this wave mode is not suitable for liquid droplet tests. To extend the application of SAW sensing from gaseous environments to liquid droplet phases, the utilization of shear-horizontal or surface horizontal (SH) SAW delay-line becomes necessary [9,10,11]. This implies that wave-guided shear-horizontal (SH) SAW applications hold the potential for expansion into the realm of biological fields, including blood testing. Such devices use a piezoelectric substrate with inter-digital transducers (IDTs) to produce the surface-localized SH waves, such as, surface skimming bulk wave (SSBW) and leaky SAW. A material layer with a slower acoustic velocity is deposited on the piezoelectric substrate to trap the acoustic energy near the surface and prevent energy loss into the bulk material. SH-SAW can be integrated with microfluidics [12] for particle manipulation and magnetic field [13] for sensitivity advancement. While SH-SAW holds promise for various applications, and recent delay-line devices designed for liquid droplet applications has improved the temperature coefficient of frequency (TCF), resonant frequency stability at the measurement temperature, by using multi-layer waveguide and matching circuits, the challenge of the limited sensing area is still the unresolved issue [9,10,11,14,15,16,17]. The pattern-less delay-line path between the input and output IDTs is usually around a few wavelengths of the SAW due to the wave attenuation in a longer distance, which may not be sufficient for various liquid types. To increase the sensing area without requiring additional engineering, a wave path was specifically designed for this study, utilizing a wavelength of 128 μm and a delay of 60 wavelengths (equivalent to 7.68 mm).

The SH-SAW device in this study was developed with a hybrid waveguide layer to achieve higher wave quality and temperature-stability for liquid sensing applications. The device comprised three layers: an ST-90°X quartz substrate, a ZnO thin film, and a thick polymer layer. ST-90°X quartz was chosen as the sensor substrate because of its stable TCF at room temperature. ST-90°X quartz generates pseudo SAWs (PSAWs), also known as leaky SAWs (LSAWs), which are surface acoustic waves with a linear combination of phase-matched decaying and radiating partial modes [18]. As a result, the signal is weak, especially for liquid applications. Therefore, a ZnO signal enhancement layer and SH wave guiding polymer layer are added to achieve the liquid phase sensing applications.

The piezoelectric enhancement layer and polymer wave guiding layer composed the bi-layer wave guide to improve the coupling factor and temperature stability of ST-90°X quartz-based SAW device for liquid-sensing applications. These layers play an essential role in the local piezoelectric particle mobility and signal quality of SH mode SAW sensors. The relation between sensitivity and the waveguide layer composition in several SH mode liquid sensors was studied [19,20,21,22].

Considering that ZnO/ ST-X quartz [23] and ZnO/ST-90°X quartz structure [24] were reported for their close-to-zero TCF characteristics at room temperature, we used a ZnO layer was used to improve the temperature–frequency stability in liquid. This type of bi-layer piezo structure is designed to produce a close or equal to zero TCF by depositing a negative TCF film on a positive TCF substrate, or vice versa. As for the TCF of ST-90°X quartz, it has been reported that leaky SAW has a positive TCF [25]. A good TCF and a higher velocity can be obtained for PSAW by combining a negative TCF ZnO film [26]. The surface-horizontal component was enhanced as well due to the bi-layer piezo structure. ZnO has been applied in various fields as acoustics sensing [27,28]. Compared with AlN, the film properties of ZnO can be easily controlled, and the growing/deposition processes can be simplified. For example, ZnO piezo film can be deposited at room temperature without substrate heating [29]. There are more advantages, such as low film stress, good adhesion with most substrates, thick film deposition up to tens of microns [30], and good biocompatibility. To confine the energy of the surface waves at the interface of the bi-piezo structure, an additional conformal polymer layer was deposited on it. Both photoresist and Parylene C were investigated as the polymer layers on the ZnO/quartz bi-piezo structure.

Previous work on ZnO on ST-quartz illustrates the dependence of surface wave velocity, propagation loss, and electromechanical coupling of three SAW modes [31]. This study demonstrated the velocity dispersion effects on the ST-X quartz between the thickness of add-on ZnO film and the wavelength of Rayleigh mode SAW (the generalized SAW). The authors concluded that the wave velocity is less affected by the mass loading of the ZnO film for longer wavelength designs. The mass effects from IDT electrodes were also studied for wave velocity and signal attenuation [32,33]. The researchers suggested that a longer acoustic wavelength would lower the loading impacts from the electrode mass. Therefore, a long wavelength was chosen in this research to eliminate the velocity dispersion by the add-on thin film. The ratio of the ZnO film thickness (300 nm) to the acoustic wavelength (128 μm) was around 0.0023 for the testing device in the following sections. The coupling factor for the PSAW mode increased with the presence of ZnO film. The differences in propagation loss level for layer-by-layer waveguide measurements are recognizable. The benefits of this novel ZnO plus Parylene C bi-layer waveguide are demonstrated in both numerical and experimental, which showed the signal enhancement effects from ZnO as well as the SH waves guiding effects from the Parylene C top layer. Two null frequencies showed the potential to sense liquid viscosity variation. This is the first paper that presents the signal quality enhancement for a long-wavelength SAW delay-line device with a bi-layer waveguide.

This device opens the potential for real-time blood drop viscosity test. The novelty of this research includes: (1) signal enhancement by the ZnO thin film, (2) split-electrode IDTs for ST-quartz SAW, (3) polymer waveguide for surface transverse modes confinement, and (4) the null frequency responses for liquid viscosity sensing.

## 2. Experiment

### 2.1. Sensor Configurationn

Two-port SAW devices were used with identical designs for the input and output IDTs. The delay-line is characterized by the propagation path between two IDTs. An IDT includes two sets of comb electrodes; one set of electrodes is connected to the ground and the other to the core of a coaxial cable. The surface wave speed is obtained by the SAW device’s resonant frequency times the distance from the center point of the input IDTs to the center point of the output IDTs. The Rayleigh wave is an out-of-plane surface transverse wave mode where local particles are moving in elliptical trace, and it can be generated on the surface of a ST-cut quartz. The Rayleigh wave speed of ST-cut quartz is 3158 m/s [34,35], and the surface-horizontal (SH) wave speed of ST-90°X quartz around 5070 m/s [36,37]. The fundamental resonance occurs when the excitation frequency is equal to *v*/*λ*, where *λ* is wavelength, which is the same as the periodicity *p* of IDT strips. For a basic IDT pattern, the metallization of one period is 50%, and the width of metal strips *d* is the same as the empty spacing.

The specifications for the devices in this study are listed in Table 1. Split-electrode configuration is chosen for all the IDTs of SH-SAW or PSAW devices. The aperture was set for 20 wavelengths. Wider aperture occupies more space and brings more challenges to fabrication processes since a small defect could easily short-circuit the device.

### 2.2. Fabrication

**Figure 1:**
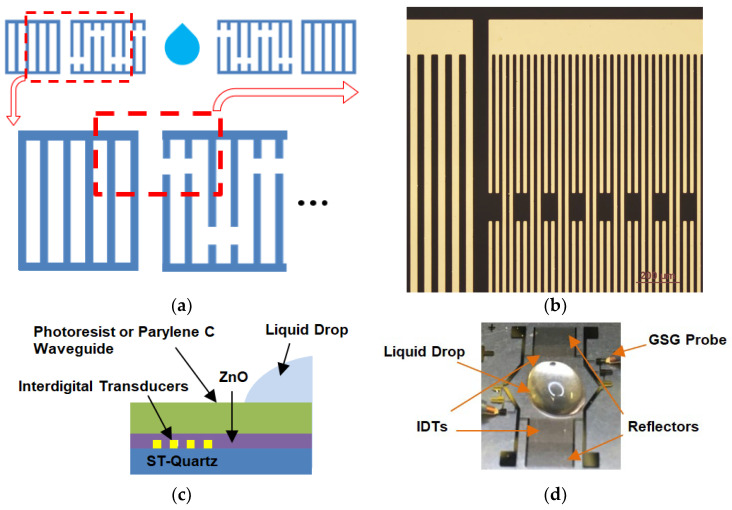
The SAW device with split-electrode transducers and reflectors on ST-90°X quartz: (**a**) Top view schematic of a split-electrode IDTs delay-line consists of an input transmitter IDTs, an output receiver IDTs, and closed reflected gratings at both ends for wave energy recovery. (**b**) Top magnified view of a fabricated device after lift-off process includes part of a split-electrode IDT and reflected gratings. (**c**) The schematic cross-section of a SH-SAW device with the bi-layer waveguide. (**d**) The DI-water droplet was placed on the delay-line path, and the device was measured through GSG probes connected to a probe station.

The lift-off process started with photolithography by creating an undercut profile. The photoresist AZ5214E (Clariant Corporation, Somerville, NJ, USA) was exposed to UV light using a laser-printed photomask (FineLine Imaging, Colorado Springs, CO, USA). The unexposed photoresist was developed in Remover PG (MicroChem, Newton, MA, USA). Afterwards, Chromium (Cr) and gold (Au) layers were deposited using thermal evaporation. The thickness of Cr and Au film were approximately 15~20 nm and 400 nm, respectively. Following the deposition, the sample was dipped in acetone to lift the photoresist layer and the metal film on top of it. The steps of metal deposition and photolithography patterning can be reversed. The metal electrode pattern can be created after wet etching. The sample was dipped in the Au etchant and Cr etchant sequentially to etch out the exposed metal area. The etching time is critical because over-etching will decrease the electrode width, which might decrease the coupling between the electrode and the piezoelectric substrate. The lift-off method does not have the electrode thinning issue. The delay-line schematic is shown in Figure 1a, and the fabricated device by the lift-off process is shown in Figure 1b, and the dimensions of the IDTs are addressed in Table 1. The cross-section is shown in Figure 1c.

The photolithography method was utilized again to define areas for the deposition of the ZnO piezoelectric film. The ZnO film was formed during the oxygen bombardment at the zinc target. The ZnO deposition was performed in custom-made RF-sputtering equipment developed at the City College of New York. A 3-inch target of pure Zn 99.995% (PURE TECH Inc., Montclair, NJ, USA) was installed on the cathode. A power of 200 W (pulse DC sputtering, ENI RPG50 generator) was used to reactively sputter Zn only using oxygen gas (O_2_) at a pressure of 15 mTorr. Argon was not employed during the sputtering process. The deposition was performed without external heating, but the sample reached a temperature of nearly 150 °C by the completion of the deposition. The deposition rate was close to 10 nm/min. The sample was then dipped in acetone to lift-off unwanted ZnO and photoresist. The film thickness was measured by an ellipsometer (Scientific Computing International FilmTek 3000SE, Carlsbad, CA, USA) with an accuracy of up to 1 nm. A surface scanning profilometer (Veeco Dektak 150, Plainview, NY, USA) was used to measure the electrode thickness and double confirmed the piezofilm thickness. The top 2.5 μm AZ-5214E photoresist (PR) waveguide layer, as well as a protection layer for the ZnO film, was spin-coated on the SAW delay-line propagation path, and unwanted photoresist was exposed and developed. The device was hard-baked for 5 min on a 110 °C hot plate to solidify the photoresist material.

Parylene C, a type of poly(p-xylylene) polymers, was used as a replacement top polymer waveguide in the later liquid drop test and was a necessary conformal layer to prevent interactions from liquids, chemical damage, and dielectric loss. The PDS 2010 (Specialty Coating Systems Inc., Indianapolis, IN, USA) was used for Parylene C deposition. Figure 1d presents a device with ZnO and photoresist waveguide and a liquid drop on the waveguide path for tests. The fabrication flow chart is included in Supplement S1.

The device was tested using a probe station (Cascade Microtech, Inc., Beaverton, OR, USA) connected to a network analyzer (Agilent Technologies, Inc., Santa Clara, CA, USA). The network analyzer and probe station network were calibrated beforehand to eliminate the system interference. The S21 signal measurements from network analyzer represent the energy loss from the emitting IDTs to the receiving IDTs. The transmission loss is minimal as the RF signal is at the same frequency as the resonant frequency of the device.

## 3. Results and Discussion

A thin film of Parylene C was used as a waveguide as well as a conformal coating to protect the delay-line device. Photoresist was replaced by Parylene C as the waveguide material. Although photoresist is an ideal material to investigate the waveguide effect, it is not ideal for liquid sensing.

The device testing and characterization data are disclosed in this section. A liquid droplet was placed on the delay-line path of the SH-SAW device with the Parylene C/ZnO bi-layer waveguide. The initial presentation in this section focuses on the results of the viscosity test conducted with a completed device. This is then followed by a detailed analysis of the layer-by-layer signal history (in this context, history means the testing of the same device, the identical IDT, every time after each layer was deposited) and simulation data. Lastly, a comprehensive summary of the three pivotal factors crucial for this SH-SAW device development is provided.

### 3.1. Liquid Droplet Experiments on SAW

Significant signal improvement from the case of 300 nm ZnO to the case of 300 nm ZnO + 3.5 μm Parylene C is shown in Supplement S2. The shift of resonant frequency was around 1.3 MHz. The lowest trough of an acoustic wave frequency scan is the lower null frequency *f_L_*, and the 2nd lowest trough is the higher null frequency *f_R_*. As the waveguide layer becomes thicker, the main lobe and side lobes become clearer.

**Figure 2:**
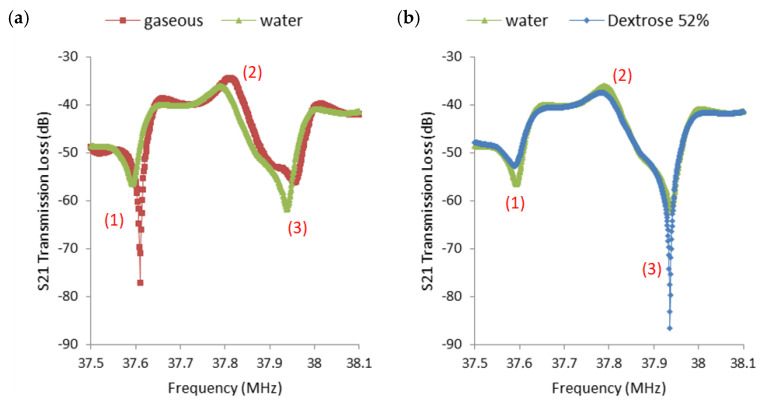
Liquid droplet experiments on a SH-SAW with Parylene C/ZnO bi-layer waveguide to show the sensor response from gaseous (air above the Parylene 3.5 μm layer) to water to viscous liquid: (**a**) A 40 μL deionized (DI) water drop deposited on the propagation path compared to gaseous (air) condition. The two null frequencies (location (1) and (3)) exhibit distinct responses to variations in the conditions. (**b**) A drop of 40 μL dextrose-water mixture (weight ratio DI−water: dextrose powder = 1:0.52) compared to a 40 μL deionized water drop. The loss decreases at location (1) while it increases at location (3). The response at location (2) exhibits subtle changes in response to variations in the conditions.

The liquid drop test was demonstrated in Figure 2a using the same device with 300 nm ZnO + 3.5 μm Parylene C waveguide as Supplement S2. A drop of 40 μL DI-water was deposited on the delay line path using a micro-pipet. The transmission loss at the lower null frequency *f_L_* (location (1)) decreased from −77.19 dB to −56.56 dB, and the transmission loss at the higher null frequency *f_R_* (location (3)) increased from −56.29 dB to −61.83 dB. The transmission loss at *f_L_* varied more than 20 dB from air to DI-water. The same change in frequency response due to liquid drop can also be observed (see Supplement S5). As the viscosity/density of the liquid increased, the *f_L_* and *f_R_* behaved in an opposite fashion, as shown in Figure 2b. The transmission loss at *f_R_* varied by more than 25 dB from DI-water to 52% dextrose.

The ladder style is adopted in our delay-line design, excluding the obstacle-free propagation path between two IDTs. The ladder-type filter is well known in one-port SAW resonators and filters on ST-quartz [38,39]. A typical ladder structure includes an IDT and reflectors at both ends. The long reflector gratings at both ends of the delay-line structure offer sharp null frequencies, as shown in Figure 2. A basic inductance and capacitance (LC) equivalent circuit for a general ladder-type SAW device includes one series arm and one parallel arm [38]. The resonant frequencies of both arms are slightly different. Due to the nature of in-phase wave addition, the S21 response offers a plateau region between two null frequencies for ladder-type SAW devices. Such a characteristic was well captured in the presented SH mode device. The resonant frequency of the series-arm was smaller than that of the parallel-arm. The first sharp null frequency, *f_L_*, in Figure 2a,b location (1), was directly related to the insertion effect of the inductor value into the series-arm, and the second sharp null frequency, *f_R_*, in Figure 2a,b location (3), was directly related to the insertion effect of the inductor value into the parallel-arm. Here, it could be stated that the loading change from dry (air) to liquid had significant effects on *f_L_* (see Supplement S5), and the combination of viscosity and density from the water-like liquid has clear effects on *f_R_*. The transmission loss and frequency information at the three locations are listed in Supplement S3.

From the presented result, the ZnO film improved the local particle mobility and signal intensity. The liquid drop test validated the existence of the surface-horizontal SAW. It was known that the polymer waveguide thickness would affect the SH wave signal quality. With 300 nm ZnO film, the total waveguide thickness can be significantly decreased for long-wavelength ST-quartz SH mode devices. With the conformal waveguide layer and ZnO layer, the device is capable for liquid drop applications. The two lowest troughs of an acoustic wave frequency scan can be the indicator for dry to liquid drop condition and monitor for kinetic viscosity. This SH mode device could be applied for real-time blood viscosity detection.

**Figure 3:**
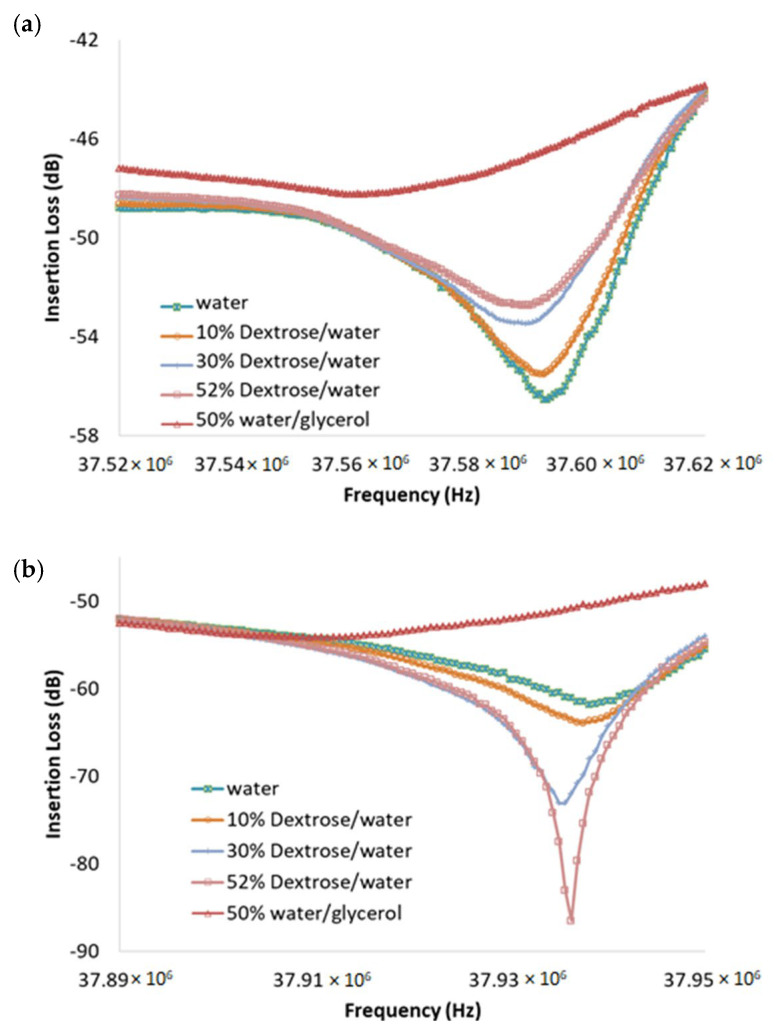
The frequency response for five liquid drops, one deionized water, three dextrose/deionized water weight ratio mixtures (10%, 30%, 52%), and a 50% deionized water/glycerol weight ratio mixture: (**a**) At the lower null frequency *f_L_* (location (1) of Figure 2), the loss decreases as the viscosity of the dextrose/water mixture increases. The wave signal becomes less pronounced for higher viscosity liquid. (**b**) At the higher null frequency *f_R_* (location (3) of Figure 2), the loss increases as the viscosity of the dextrose/water mixture increases. The wave signal becomes more pronounced. However, the water/glycerol mixture badly dampened the signal.

**Figure 4:**
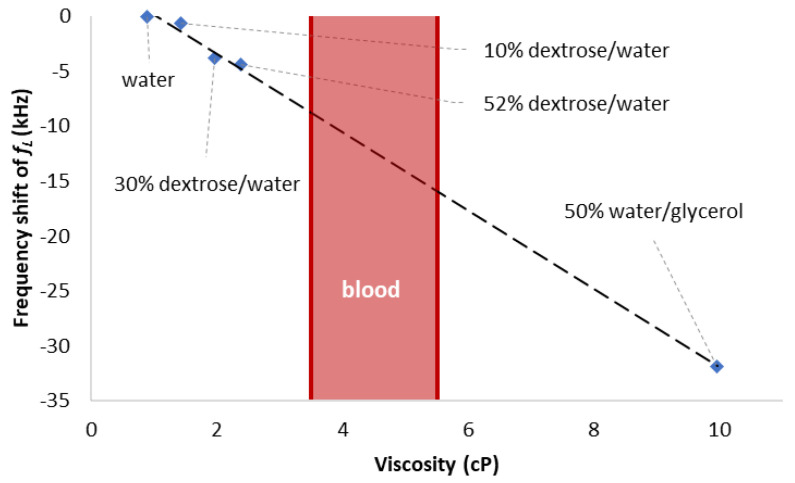
The relationship between viscosity and *f_L_* frequency shift of all four mixtures with respect to water. Blood viscosity is between 3.5 cP and 5.5 cP. The dotted line acted as an indicator for the trend between viscosity and frequency shift. The experiment data show the sensing capability within the blood viscosity range.

The viscosity test was continued for several dextrose/DI-water mixtures. The tested dextrose/DI-water weight ratios were 10%, 30%, 52%. A higher weight ratio percentage of dextrose means a higher viscosity. The response of the lower null frequency, *f_L_* (Figure 2 location (1)), reacts in the opposite fashion compared to *f_R_* (Figure 2 location (3)). Those three dextrose/ DI–water mixtures were also tested using ARES-G2 SN4010-0337 Rheometer under 10 s^−1^ shear rate, since the shear rate in human in veins is around 10 s-1 under normal physiology flow conditions [40]. The mean value from each experiment condition was applied to the Andrade equation to obtain an estimated dynamic viscosity value at 25 °C room temperature. The two constants in the Andrade equation were calculated based on two experimental data points. The estimated dynamic viscosity values by ARES-G2 rotational rheometer at 25 °C room temperature for 10%, 30%, 52% dextrose/DI–water mixtures are 1.428 cP, 1.96 cP, and 2.385 cP, respectively. Since blood viscosity is usually considered between 3.5 cP and 5.5 cP [1], another higher viscosity test is needed. A mixture of 50% DI-water/glycerol weight ratio was used to the tested. The evaluated viscosity value based on ARES-G2 SN4010-0337 Rheometer test is 9.966 cP. The liquid drop test at location (1) and (3) are shown in Figure 3. The result shows that the device has the potential to detect the viscosity change in human blood. The relationship between viscosity and *f_L_* frequency shift for all four mixtures with respect to water is shown in Figure 4. The slope of the linear trend line indicates that the sensitivity of the device is 3.57 kHz shift per centipoise, and the 625 Hz frequency spacing between data points is the resolution of the measurements. The viscosity range of blood is shown in the red band.

### 3.2. ZnO Enhancement Layer and Photoresist Waveguide Layer

#### 3.2.1. Photoresist/ZnO as the Waveguide Layer

**Figure 5:**
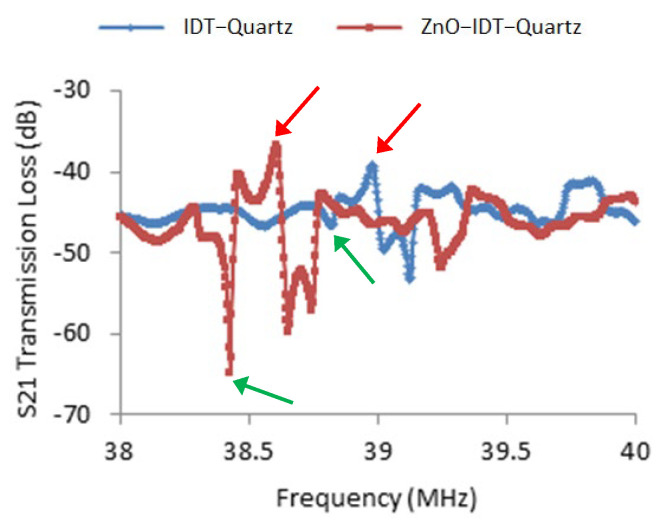
S21 history of a SAW delay-line device with/without 300 nm ZnO enhancement layer (Table 1). The difference of the dB level from the peak (red arrows) of the main lobe to the left trough (green arrows) of the main lobe improved from 7.519 dB to 28.111 dB. The ZnO enhancement effect can be directly observed.

The layer-by-layer signal history demonstration includes the device without the waveguide layer, the device with the waveguide layer of 300 nm ZnO thin film, and finally the device with the composite waveguide layer of 300 nm ZnO and 2.5 μm photoresist. Figure 5 shows a dramatic signal improvement from 300 nm ZnO film. Relative side lobe level (RSLL), the amplitude at the peak of the main lobe to the amplitude at the peak of a side lobe, improved by 224% from 2.637 dB to 5.902 dB. The difference of the dB level from the peak of the main lobe to the left trough of the main lobe improved from 7.519 dB to 28.111 dB. This evidence directly shows the increasing displacement for local particles. As more material was added on top of the quartz substrate, the resonant frequencies decreased. The arrow indicates the same resonant for each device status. A layer of photoresist was coated on the same device as a waveguide to fulfill surface-horizontal wave mode, and the S21 signal is shown in Supplement S4.

**Figure 6:**
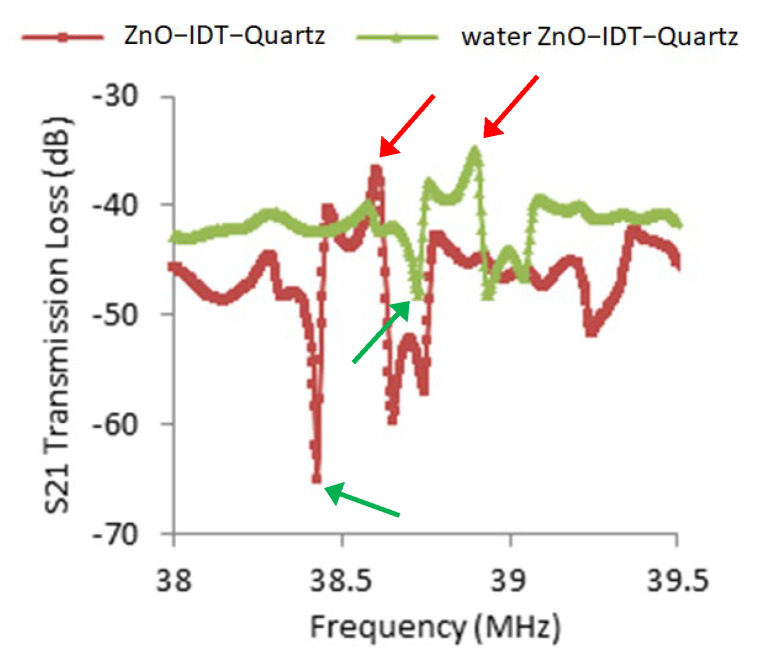
Graph representing the 40 μL DI−water drop test of a ZnO/IDT/quartz device (Table 1). The difference in the dB level from the peak (red arrows) of the main lobe to the left trough (green arrows) of the main lobe decreased from 24.605 dB to 10.394 dB. The damping effect by the water droplet showed the existence of out-of-plane modes, and the current device still generated a low percentage of SH mode waves.

The liquid test for the device with waveguide layers validated the intensity of the SH wave mode. Figure 6 shows that water dampens the device while no polymer layer is present. The high percentage of radiating partial modes remain. Therefore, when a water drop is present, the main lobe becomes less pronounced. The difference of the dB level from the peak of the main lobe to the left trough of the main lobe decreased from 24.605 dB to 10.394 dB. In Supplement S5, as the same device is coated with an extra photoresist top layer, the main lobe signal remains stable when a water drop is present. The signal comparison of before and after a 40 μL DI-water drop on the photoresist-coated propagation path shows that the wave signal is preserved. The signal quality was retained as decaying modes dominated. Up to this moment, this SH-SAW device has been established.

The frequency shift of Pseudo-SAW (SH mode) was demonstrated due to the mass loading from the additional layers, such as thick IDT electrodes, 300 nm zinc oxide layer, and photoresist waveguide layer. Additionally, SH wave phenomena are presented, where the liquid damping did not reduce the signal quality, and the wave propagated at the interface of the waveguide and double piezoelectric structure. Compared to the result in our early work [41], the photoresist layer decreased from 3.6 µm to 2.5 µm with the help of the ZnO enhancement layer, and the total structure thickness has reduced.

#### 3.2.2. ZnO Enhancement Layer

The contribution from the ZnO enhancement layer was not directly proven in the previous experimental data. Therefore, another device was used to test the measurements for photoresist only and photoresist on ZnO. The experimental measurements shown in Supplement S6 provided direct evidence of the benefits from the ZnO layer with the same photoresist thickness. The signal improvement from the combined guiding layer was demonstrated, and the wave signals of each condition were shown separately. The measurements were all from the same SAW device, but only the top layers were modified.

After coating the photoresist waveguide, the bulk waves were damped by the viscoelastic layer, and only SH modes were preserved. The purpose of this study was to eliminate the device difference and directly prove the signal enhancement by the double-piezo-layered PSAW device.

### 3.3. Simulation

Finite element analysis (FEA) simulation with COMSOL Multiphysics was used to model the bi-layer SH-SAW device to obtain the wave mode and the frequency shift for each added waveguide layer. Two models were built to perform this analysis. First, a unit IDTs cell model on ST-90°X quartz was established to verify the mode shape and obtain the resonant frequency. Second, a delay-line model was utilized to verify the frequency shift trend for each added waveguide with the experimental data. The ZnO enhancement effect was observed in both models and the displacement field of local particles showed that the X component is 10 orders larger than the other two directions.

#### 3.3.1. Unit IDTs Cell Model on ST-90°X Quartz

**Figure 7:**
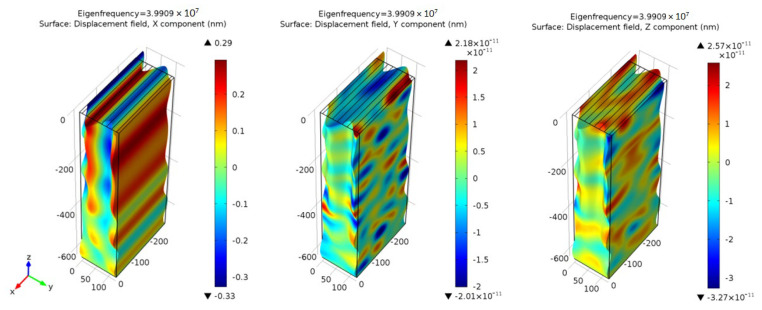
The individual displacement field of the X, Y, and Z components and the eigenfrequency of the wave mode. The results show the horizontal wave mode and X is the dominant particle moving direction. The displacement value is 10 orders larger than the Y and Z components.

COMSOL Multiphysics was used to analyze the eigenfrequency for the transverse horizontal wave mode in XY-plane around the pseudo SAW resonant frequency and the displacement response in the frequency domain. The simulation was employed for the validation of the experimental data and literature predictions. For this COMSOL Multiphysics simulation, a 3D single-cell model was created, and the solid mechanics and static electrics boundary conditions were applied. The simulation in Figure 7 is based on a unit IDTs set without any over layers. The mesh size control for the convergence test is performed to obtain a reasonable element size for the unit-cell models. The element size in all simulations remains at a similar level. The results show the horizontal wave mode and the displacement field of the three components, where X is the dominant direction.

The result of the modal analysis for the Au electrode on ST-90°X quartz identified the eigenfrequency and the displacement mode shape for horizontal transverse waves around the resonant frequency at 39.909 MHz, as Figure 7. The resonant frequency was higher than the theoretical and experimental values, due to the limited single IDT cell model size and less restricted boundary conditions. The particle displacement in the X direction was around ten orders larger than the displacement in the two other directions. The waves propagated in the Y direction, and the main displacement was in the X direction. One full horizontal transverse wave can be observed within the single-cell model. The horizontal transverse mode along the thickness direction could also be observed within the five wavelengths (640 μm) depth model. Although there were waves located deeper in the substrate, the wave propagation direction remained the same. Most waves do not propagate or penetrate in the Z direction.

The displacement plots of the ZnO enhancement layer for two cutlines are shown in Supplement S7. One cutline is at the center symmetric line of IDTs parallel to x axis on the top quartz surface, and the other is the thickness penetration cutline parallel to the z axis at the center of the geometry. The ZnO layer is on the top of Au IDTs and the substrate. Three ZnO thicknesses, 400 nm, 600 nm, and 800 nm, were analyzed to determine the optimal layer parameter. The three thicknesses showed little variation for the X displacement component, and its value was around 11 orders larger than the other two displacement components. Transverse horizontal waves still dominated the wave behavior with a zinc oxide layer in this single-cell model. The thickness of the piezoelectric thin layer is not an important factor from this analysis. Both displacement value and eigenfrequency remained close between three cases. The 400 nm zinc oxide thickness was used for all the following simulations.

The results in Supplement S7 demonstrate the mobility improvement for local particles at the top surface of the quartz substrate with the zinc oxide over layer, the X component of particles along both the surface propagation direction and thickness direction increase. The X component (radiating partial modes) does not show energy decade within the thickness of five wavelengths with or without a ZnO layer. Scientists applied thicker polymer waveguides [21,22,28,42] to guide the waves and decrease the radiating partial modes.

#### 3.3.2. Simulation of Displacement Field in Frequency Domain with a Delay Line Model

The previous single-cell model provided the mode shape and eigenfrequency information. A frequency span analysis was carried out in this section. Due to the computation efficiency, a thin-wall delay line model was built to analyze the displacement field response in the frequency domain. This analysis provided information about the eigenfrequency shift after each over layer to verify the trend with experiment data.

The response demonstrated the change from bare quartz to the final three-layered structure. The frequency domain analysis showed the same resonant frequency as the eigenfrequency analysis. This delay-line model was focused on the frequency domain to reveal the total displacement (the absolute value from the three X, Y, and Z components) field response. The total displacement field response for the delay-line model from bare ST-90°X quartz substrate with Au IDT, ZnO on the IDT/quartz, and Parylene C on the ZnO/IDT/quartz are shown in Supplement S8. The wave speed obtained from the simulation data was close to the values in the literature [36,37].

These simulation results concluded that the ZnO wave guiding layer had greatly enhanced the dielectric coupling of the device. Then, the displacement at the resonant frequency increased one order of magnitude with the help of the polymer layer, and the neighboring peaks were suppressed. More wave modes were pronounced after the ZnO deposition. The resonant frequency of PSAW and its neighboring resonant frequencies were enhanced due to the ZnO growing orientation. Then, the out-of-plane modes were eliminated by the polymer waveguide. This further proved that the polymer waveguide was necessary to fulfill the SH mode device. The frequency shifts due to the additional 1.5 μm Parylene C layer were consistent with the experimental results shown in Table 2. The same device was first coated with 1.5 μm Parylene C and then further coated with the second layer of Parylene C to reach a total of 3.5 μm thickness. The experimental and numerical results a showed strong signal response and local displacement field with the Parylene C/ZnO bi-layer waveguide structure on top of the delay-line structure and provided the potential application for the SH mode device to perform the real-time liquid drop test.

### 3.4. Device Development

This SH-SAW device development includes IDT design and allocation; microfabrication; device test and characterization; simulation and validation. The Parylene C/ZnO bi-layer waveguide is a significant breakthrough during the development process. This section provides a summary of the three key findings to successfully establish a SH-SAW device on the ST-90°X quartz with a long delay-line path: split IDTs, ZnO enhancement layer, and the top polymer waveguide.

- The split interdigital transducers eliminate phase interference from triple-transit-interference (TTI).

Due to low-piezoelectric coupling for ST-quartz and spurious bulk waves at the electrode boundaries within long repetitive IDTs, the traveling waves generated by single electrode IDTs degrade or leak quickly due to destructive wave interactions. This is known as triple-transit-interference (TTI), which is due to multiple reflections between input and output IDTs. By constructing splitting electrodes, the out-of-phase reflection waves will cancel out from each other. Our previous study [41] showed the 3 dB bandwidth at the main lobe narrowed down around 3.38 times comparing split-electrodes to single-electrodes.

- The signal enhanced by adding thin ZnO layer on ST-quartz.

The ZnO thin film enhanced the wave signal as well as local particle mobility. The relative side lobe level (RSLL) improved by 224% from 2.637 dB to 5.902 dB in Figure 5. The wave amplitude on the quartz substrate surface in the single-cell model exhibited a three-fold increase in the direction of wave propagation, as demonstrated in Supplement S7 Figure S7-1. Both experimental and simulation results showed the increase in displacement from local particles. However, the top Parylene C layer was still needed afterward to obtain a decaying mode dominated SH-SAW device.

- The water drop on the ZnO/IDT device shows that both standing waves and horizontal waves are present. By adding on a polymer layer, most waves move toward the horizontal plain.

In Figure 6, the dB value for the left peak of the main lobe to the left trough of the main lobe decreased from 24.61 dB to 10.39 dB when the water dropped directly on ZnO film without a polymer waveguide. The signal quality decreased, as the out-of-plain waves were dampened by the water drop. In Supplement S5 Figure S5, while the polymer waveguide was present, the dB value for the left peak of the main lobe to the left trough of the main lobe remained larger than 16 dB, and the wave signal quality was maintained. A SH-SAW on ST-90°X quartz with the Parylene C/ZnO bi-layer waveguide has been well established.

## 4. Conclusions

This paper presents the design and characterization of a novel bi-layer waveguide for a SH-SAW device with a 7.68 mm sensing area specifically tailored for biosensing applications such as blood viscosity monitoring. The extended sensing area offers increased flexibility for sample preparation and accommodates a wide range of liquid viscosities.

FEA simulation results verify that the X component is the dominant displacement direction, and the ZnO layer improves the particle mobility in all directions.

The novelty of this research includes the following: (1) signal enhancement by a ZnO thin fil; (2) elimination of bulk wave interference with split-electrode IDTs; (3) confinement of surface transverse modes using a polymer waveguide; and (4) liquid viscosity sensing through the direct response of the null frequencies.

This research presents signal quality enhancement with a long-wavelength SH-SAW device with a sensitivity of 3.57 kHz shift per centipoise. The null frequency can be determined within ±0.3125 kHz. Therefore, the viscosity precision is known to ±0.0875 cP. With additional measurements, precision of the order of ±0.01 cP is possible. This device demonstrates the potential for high-precision blood viscosity monitoring.

## Figures and Tables

**Table 1:** Specifications of IDTs.

**Device Parameter**	**Specification**
Wavelength	16 μm × 8 (split-electrode design)
Input/output IDT set numbers	20/10
Overlapping aperture	20 λ
Delay-line path length	60 λ
Aperture of dummy strips	5 λ
Space between reflector and IDT	16/2 + 16 × 4 μm
Reflector numbers	60/40 strips
Au thickness	380~400 nm
ZnO thickness	300 nm

**Table 2:** Cases of Parylene C/ZnO Waveguides.

Simulation Cases	Resonant Frequency (MHz)	Frequency Shift (MHz)
400 nm ZnO/IDT/quartz	39.174	0
1.5 μm Parylene C/400 nm ZnO/IDT/quartz	39.039	0.135
**Experiment cases** (Supplement S2)	**Resonant frequency (MHz)**	**Frequency shift (MHz)**
300 nm ZnO/IDT/quartz	38.808	0
1.5 μm Parylene C/300 nm ZnO/IDT/quartz	38.669	0.139
3.5 μm Parylene C/300 nm ZnO/IDT/quartz	37.810	0.997

## Supplementary Materials

The following supporting information can be downloaded at: https://www.mdpi.com/article/10.3390/s23135911/s1, Supplement S1–S8.

## References

[1] Nader E., Skinner S., Romana M., Fort R., Lemonne N., Guillot N., Gauthier A., Antoine-Jonville S., Renoux C., Hardy-Dessources M.-D. (2019). Blood Rheology: Key Parameters, Impact on Blood Flow, Role in Sickle Cell Disease and Effects of Exercise. Front. Physiol..

[2] Kwaan H., Bongu A. (1999). The Hyperviscosity Syndromes. Semin. Thromb. Hemost..

[3] Almarshad H.A., Hassan F.M. (2016). Alterations in Blood Coagulation and Viscosity among Young Male Cigarette Smokers of Al-Jouf Region in Saudi Arabia. Clin. Appl. Thromb. Hemost..

[4] Wang W., Xue X., Fan S., Liu M., Liang Y., Lu M. (2020). Development of a Wireless and Passive Temperature-Compensated SAW Strain Sensor. Sens. Actuators A Phys..

[5] Shu L., Wang X., Li L., Yan D., Peng L., Fan L., Wu W. (2019). The Investigation of Integrated SAW Strain Sensor Based on AlN/TC4 Structure. Sens. Actuators A Phys..

[6] Lamanna L., Rizzi F., Bhethanabotla V.R., De Vittorio M. (2020). GHz AlN-Based Multiple Mode SAW Temperature Sensor Fabricated on PEN Substrate. Sens. Actuators A Phys..

[7] Li D., Zu X., Ao D., Tang Q., Fu Y., Guo Y., Bilawal K., Faheem M.B., Li L., Li S. (2019). High Humidity Enhanced Surface Acoustic Wave (SAW) H2S Sensors Based on Sol–Gel CuO Films. Sens. Actuators B Chem..

[8] Ye X., Fang L., Liang B., Wang Q., Wang X., He L., Bei W., Ko W.H. (2011). Studies of a High-Sensitive Surface Acoustic Wave Sensor for Passive Wireless Blood Pressure Measurement. Sens. Actuators A Phys..

[9] Nomura T., Saitoh A., Miyazaki T. (2003). Liquid Sensor Probe Using Reflecting SH-SAW Delay Line. Sens. Actuators B Chem..

[10] Sehra G., Cole M., Gardner J.W. (2004). Miniature Taste Sensing System Based on Dual SH-SAW Sensor Device: An Electronic Tongue. Sens. Actuators B Chem..

[11] Martin F., Newton M.I., McHale G., Melzak K.A., Gizeli E. (2004). Pulse Mode Shear Horizontal-Surface Acoustic Wave (SH-SAW) System for Liquid Based Sensing Applications. Biosens. Bioelectron..

[12] Winkler A., Brünig R., Faust C., Weser R., Schmidt H. (2016). Towards Efficient Surface Acoustic Wave (SAW)-Based Microfluidic Actuators. Sens. Actuators A Phys..

[13] Elhosni M., Elmazria O., Petit-Watelot S., Bouvot L., Zhgoon S., Talbi A., Hehn M., Aissa K.A., Hage-Ali S., Lacour D. (2016). Magnetic Field SAW Sensors Based on Magnetostrictive-Piezoelectric Layered Structures: FEM Modeling and Experimental Validation. Sens. Actuators A Phys..

[14] Kanouni F., Amara S., Assali A., Arab F., Qin Z. (2020). A P-Matrix-Based Model for the Frequency Analysis of IDT/AlScN/Sapphire SAW-Delay Line. Sens. Actuators A Phys..

[15] Achour B., Attia G., Zerrouki C., Fourati N., Raoof K., Yaakoubi N. (2020). Simulation/Experiment Confrontation, an Efficient Approach for Sensitive SAW Sensors Design. Sensors.

[16] Tian Y., Li H., Chen W., Lu Z., Luo W., Mu X., Wang L. (2021). A Novel Love Wave Mode Sensor Waveguide Layer with Microphononic Crystals. Appl. Sci..

[17] Choudhari A., Rube M., Sadli I., Sebeloue M., Tamarin O., Dejous C. (2022). Love Wave Acoustic Sensor Response in High Turbidity Liquid Environment. Proceedings of the Sensor 2022.

[18] Lim T.C., Farnell G.W. (1969). Character of Pseudo Surface Waves on Anisotropic Crystals. J. Acoust..

[19] Zhang F., Li S., Cao K., Wang P., Su Y., Zhu X., Wan Y. (2015). A Microfluidic Love-Wave Biosensing Device for PSA Detection Based on an Aptamer Beacon Probe. Sensors.

[20] Josse F., Bender F., Cernosek R.W. (2001). Guided Shear Horizontal Surface Acoustic Wave Sensors for Chemical and Biochemical Detection in Liquids. Anal. Chem..

[21] Mitsakakis K., Tsortos A., Kondoh J., Gizeli E. (2009). Parametric Study of SH-SAW Device Response to Various Types of Surface Perturbations. Sens. Actuators B Chem..

[22] Kovacs G., Vellekoop M.J., Haueis R., Lubking G.W., Venema A. (1994). A Love Wave Sensor for (Bio)Chemical Sensing in Liquids. Sens. Actuators A Phys..

[23] Talbi F.S.A. Zero TCF ZnO/Quartz SAW Structure for Gas Sensing Applications. Proceedings of the 2004 IEEE International Frequency Control Symposium and Exposition.

[24] Kadota M. (2000). Surface Acoustic Wave Properties of Zinc Oxide Film on Quartz Substrate. Electron. Commun. JPN.

[25] Naumenko N.F., Didenko I.S. Leaky Wave Propagation in Layered Structures. Proceedings of the 1998 IEEE Ultrasonics Symposium, Proceedings (Cat. No. 98CH36102).

[26] Kadota M. (1997). Combination of ZnO Film and Quartz to Realize Large Coupling Factor and Excellent Temperature Coefficient for SAW Devices. Proceedings of the 1997 IEEE Ultrasonics Symposium Proceedings, An International Symposium (Cat. No.97CH36118).

[27] Li S., Wan Y., Fan C., Su Y. (2017). Theoretical Study of Monolayer and Double-Layer Waveguide Love Wave Sensors for Achieving High Sensitivity. Sensors.

[28] Newton M.I., McHale G., Martin F. (2004). Experimental Study of Love Wave Devices with Thick Guiding Layers. Sens. Actuators A Phys..

[29] Yoshino Y. (2009). Piezoelectric Thin Films and Their Applications for Electronics. J. Appl. Phys..

[30] Fan L., Zhang S., Ge H., Zhang H. (2013). Theoretical Investigation of Acoustic Wave Devices Based on Different Piezoelectric Films Deposited on Silicon Carbide. J. Appl. Phys..

[31] Hickernell F.S., Adler E.L. The Experimental and Theoretical Characterization of SAW Modes on ST-X Quartz with a Zinc Oxide Film Layer. Proceedings of the International Frequency Control Symposium.

[32] Cambon G. (1969). Dispersive Rayleigh Waves on Quartz. Electron. Lett..

[33] Cambon G., Rouzeyre M. (1970). Attenuation of Dispersive Rayleigh Waves on Quartz. Electron. Lett..

[34] Ballantine D., White R.M., Martin S.J., Ricco A.J., Zellers E.T., Frye G.C., Wohltjen H. (1996). Acoustic Wave Sensors: Theory, Design, & Physico-Chemical Applications.

[35] Fu Y.Q., Luo J.K., Nguyen N.T., Walton A.J., Flewitt A.J., Zu X.T., Li Y., McHale G., Matthews A., Iborra E. (2017). Advances in Piezoelectric Thin Films for Acoustic Biosensors, Acoustofluidics and Lab-on-Chip Applications. Prog. Mater. Sci..

[36] Da Cunha M.P. High Velocity Pseudo Surface Waves (HVPSAW): Further Insight. Proceedings of the 1996 IEEE Ultrasonics Symposium.

[37] Hickernell F.S. (2000). Thin-Films for Saw Devices. Int. J. High Speed Electron. Syst..

[38] Hikita M. (2000). SAW Antenna Duplexers for Mobile Communication. Int. J. High Speed Electron. Syst..

[39] Satoh Y., Ikata O. (2000). Ladder Type SAW Filter and Its Application to High Power SAW Devices. Advances in Surface Acoustic Wave Technology, Systems and Applications.

[40] Sakariassen K.S., Orning L., Turitto V.T. (2015). The Impact of Blood Shear Rate on Arterial Thrombus Formation. Future Sci. OA.

[41] Lee K.-L., Voiculescu I. Study of Low-Frequency Narrow Bandwidth Surface Acoustic Wave Sensor for Liquid Applications. Proceedings of the Volume 10: Micro- and Nano-Systems Engineering and Packaging, American Society of Mechanical Engineers.

[42] McHale G., Newton M.I., Martin F., Gizeli E., Melzak K.A. (2001). Resonant Conditions for Love Wave Guiding Layer Thickness. Appl. Phys. Lett..

