# Supplementary material for: Liquid Viscosity Sensor Using a Surface Acoustic Wave Device for Medical Applications Including Blood and Plasma
Source: Sensors (Basel). 2023 Jun 26;23(13):5911. doi: 10.3390/s23135911 (PMC10346882; doi:10.3390/s23135911)
Supplement: Supplementary file 1 [file sensors-23-05911-s001.zip › sensors-2395101-supplementary.pdf]

Supplement Information

Liquid Viscosity Sensor Using a Surface Acoustic Wave Device for Medical Applications Including Blood and Plasma

Kun-Lin Lee, Glen Kowach, Fang Li, Ioana Voiculescu

S1. The lift-off process started with photolithography by creating an undercut profile. The photoresist AZ5214E (Clariant Corporation, Somerville, NJ) was exposed to UV light using a laser-printed photomask (FineLine Imaging, Colorado Springs, CO.). The unexposed photoresist was developed in Remover PG (MicroChem, Newton, MA). Afterwards, Chromium (Cr) and gold (Au) layers were deposited using thermal evaporation. The thickness of Cr and Au film were around 15~20 nm and 400 nm, respectively. Following the deposition, the sample was dipped in acetone to lift the photoresist layer and the metal film on top of it. The photolithography method was utilized again to define areas for the deposition of the ZnO piezoelectric film. The ZnO film was formed during the oxygen bombardment at the zinc target. The ZnO deposition was performed in custom-made RF-sputtering equipment. A power of 200 W (pulse DC sputtering, ENI RPG50 generator) was used to reactively sputter Zn using only oxygen gas (O2) at a pressure of 15 mTorr. Argon was not employed during the sputtering process. The deposition was performed without external heating, but the sample reached a temperature of nearly 150 °C by the completion of the deposition. The deposition rate was close to 10 nm/mins. The sample was then dipped in acetone to lift-off unwanted ZnO and photoresist. Finally, PDS 2010 (Specialty Coating Systems Inc., Indianapolis, IN) was used for Parylene C deposition.

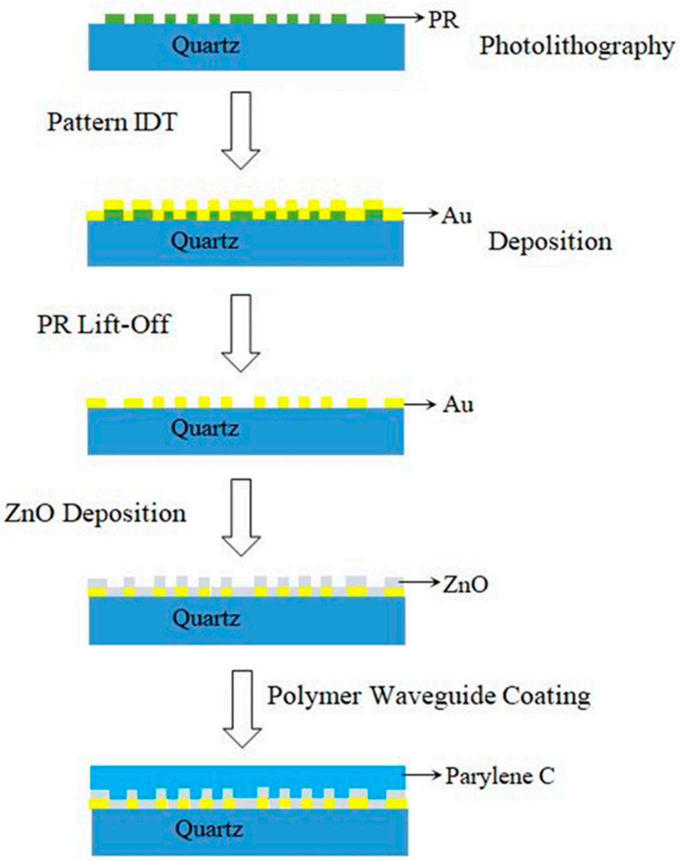

Figure S1. The fabrication process flow for the SH-SAW device with the Parylene C/ZnO bi-layer waveguide. The processes start from photolithography and lift-off process, where PR stands for photoresist, to create metal electrodes, and is then followed by the ZnO enhancement layer deposition. Finally, the device is coated with Parylene C as the top waveguide layer.

S2. After the ZnO waveguide was deposited, a thin film of Parylene C is used to protect it from humidity and direct contact with liquid drops. Parylene C is a type of poly(p-xylylene) frequently used as a conformal coating to protect electronics from moisture, chemical damage, and dielectric loss, and it is resistant to UV and thermal treatment. The SCS (Specialty Coating Systems Inc., Indianapolis, IN) was used for Parylene C deposition. We replaced photoresist with Parylene C as the finalized top waveguide material. Although photoresist is an ideal material to investigate the waveguide effect, it is less ideal for liquid drops. Figure S1 shows the signal improvement from the case of 300 nm ZnO to the case of 300 nm ZnO + 3.5 μm Parylene C. The peak of the main lobe and the two null frequencies are clearly identified with the existence of the Parylene C layer. The dB difference between the lowest null frequency and the main lobe peak improved from unidentifiable to more than 40 dB separation. The shift in resonant frequency was around 1.3 MHz. The lowest trough of an acoustic wave frequency scan is the lower null frequency f_L, and the 2nd lowest trough is the higher null frequency f_R. As the waveguide layer becomes thicker, the main lobe and side lobes become clearer.

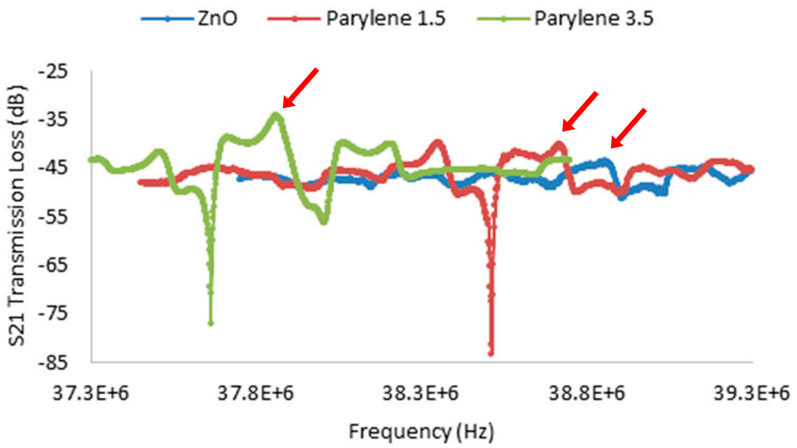

Figure S2. The signal comparison by adding Parylene C waveguide: 1.5 μm and 2 μm were deposited on the same device sequentially. The resonant frequency shifts were around 1.3 MHz from 300 nm ZnO to 300 nm ZnO + 3.5 μm Parylene C.

S3. The transmission loss values and frequencies at location (1) (2) (3) from the liquid drop test demonstrated in Figure 2 are listed in Table S3. A drop of 40 μl DI-water was deposited on the delay line path using a micro-pipet. The transmission loss at the lower null frequency f_L (location (1)) decreased from -77.19 dB to -56.56 dB, and the transmission loss at the higher null frequency f_R (location (3)) increased from -56.29 dB to -61.83 dB. The transmission loss at f_L varied more than 20dB from air to DI-water. The same change in frequency response due to liquid drop can also be observed (see Supplement S5). As the viscosity/density of the liquid increased, the f_L and f_R behaved in an opposite fashion, as shown in Figure 2(b). The transmission loss at f_R varied by more than 25 dB from DI-water to 52% dextrose.

Table S3. S21 transmission loss values and frequencies for liquid drop test at the three locations in Figure 2.

S21 (dB) / Frequency (MHz)	Parylene C (3.5 μm)	Water (~0.9 cP)	Dextrose 52% (~2.4 cP)
Location (1)	-77.19 dB / 37.61 MHz	-56.56 dB / 37.59 MHz	-52.74 dB / 37.59 MHz
Location (2)	-34.36 dB / 37.81 MHz	-36.11 dB / 37.79 MHz	-37.47 dB / 37.78 MHz
Location (3)	-56.29 dB / 37.95 MHz	-61.83 dB / 37.94 MHz	-86.51 dB / 37.94 MHz

S4. The measurements in Figure S4 showed the signal before and after the presence of photoresist (PR) waveguide layer. As majority waves moved toward surface-horizontal mode, side lobe frequencies were further pronounced. The SH mode was fulfilled by the coated photoresist layer on the same device as a waveguide. The liquid drop test in Section 3.1 was direct evidence of the existence of surface-horizontal waves.

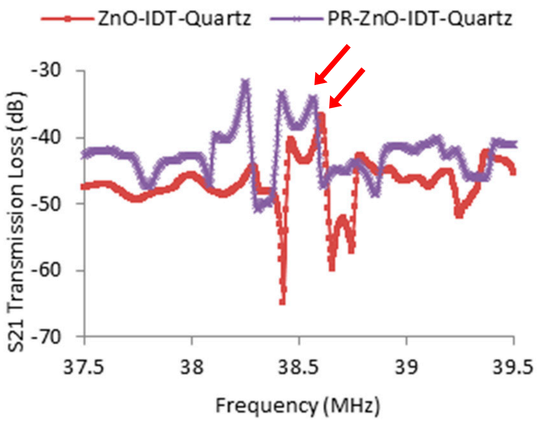

Figure S4. S21 response history of a device (Table 1) with/without 2.5 μm AZ-5214E photoresist (PR) waveguide.

S5. The measurements in Figure S5 showed a signal change due to a 40 μl water drop on the propagation path. The signal quality retained as decaying modes dominated, and the SH-SAW device is established. The dB value for the left peak to the left trough of the main lobe remained larger than 16 dB. The value for dry (air) condition is 16.69 dB and 23.01 dB for water drop condition. The distance between the left peak of the main lobe and the right peak of the left lower side lobe is 165.625 kHz apart for dry (air) condition and 178.125 kHz apart for water drop condition. The water drop did not degrade the power intensity and the surface wave formation.

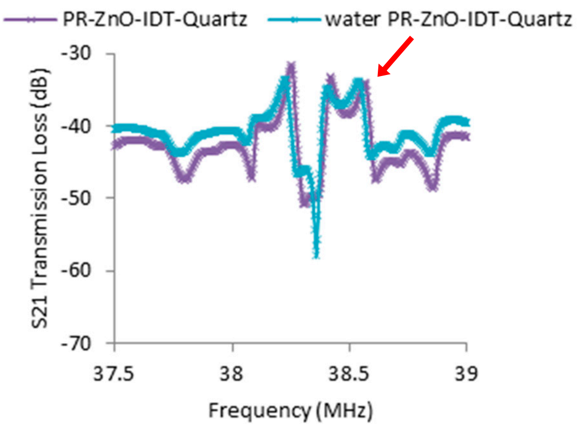

Figure S5. Graph depicting the 40 μl water drop test of a device (Table 1) on the delay-line path coated with photoresist (PR) and ZnO bilayer.

S6. We present the 2.5 μm AZ-5214E photoresist waveguide effect with and without a 300 nm ZnO layer on device from Table 1. The ZnO-free device was spin-coated, exposed, and developed to form the 2.5 μm photoresist waveguide. The top view of the SAW device is the same as Figure 1(b). After the measurement for the ZnO-free device was performed, the photoresist layer was stripped and removed. Then, a 300 nm zinc oxide layer was deposited on the propagation path and followed by another 2.5 μm photoresist waveguide. The measurements were all from the same SAW device, and only the top layers were modified to show the direct benefit from the ZnO enhancement layer.

Measurement in Figure S6-1 showed S21 measurements with and without photoresist layer. The response was cleaner for the photoresist (PR)/IDT case, which could be the damping effect by the polymer layer, so the radiating partial modes into the substrate bulk were eliminated. Measurement in Figure S6-2 showed the signal comparison of both PR/IDT and PR/ZnO/IDT cases. The main lobe was further pronounced with the both ZnO enhancement layer and PR waveguide. A 20 dB transmission loss difference from the main lobe peak to the lower null is observed, which is approximately twice the loss difference in the PR/IDT case.

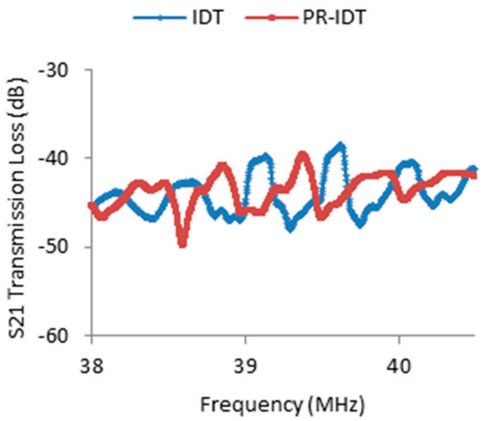

Figure S6-1. S21 signals with and without the photoresist layer on a bare IDT device. The response is cleaner for the PR/IDT case.

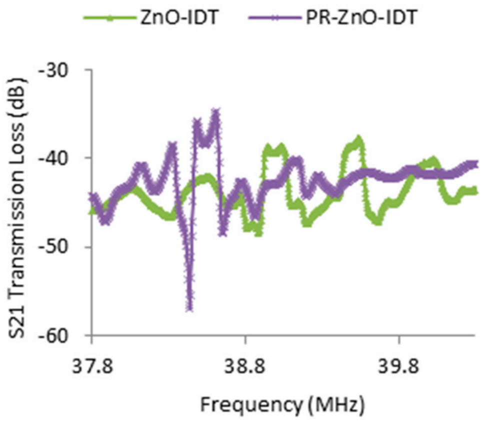

Figure S6-2. The signal with and without the photoresist layer on a ZnO/IDT device. The main lobe was further pronounced with both the ZnO enhancement layer and photoresist waveguide.

S7. The displacement plots of the ZnO enhancement layer along two cutline directions are shown here. One cutline is at the center symmetric line of IDTs parallel to the x axis on the top quartz surface, and the other is the thickness penetration cutline parallel to the z axis at the center of the geometry. These figures below are the comparison of three zinc oxide thicknesses (400 nm, 600 nm, and 800 nm): (a) The X component displacement from the surface cutline along the y axis direction on the quartz surface. (b) The X component displacement from the thickness cutline

along the z axis direction.

The amplitude of the surface waves from Figure S7-1 has increased from around 0.247 nm to 0.749 nm. The amplitude is defined by the crest and the trough in the red line region of Figure S7-1. The locations of 0 μm and 128 μm are the free boundaries, where there should be more constraints in real cases, so the two regions were not taken into consideration. The pseudo wave beneath the surface still existed within finite wavelengths (640 μm, five wavelengths) in the thickness direction, as Figure S7-2. The amplitude of the pseudo waves showed no sign of decrement along the thickness cutline of this single-cell model. The zinc oxide layer showed a significant improvement for dielectric coupling, which escalated the local displacement field and voltage potential. However, the thickness of the piezoelectric thin layer is not an important factor from this analysis. Both the displacement value and eigenfrequency remained close between the three cases. The eigenfrequency (38.949 MHz, 38.925 MHz, and 38.906 MHz, respectively, for zinc oxide thicknesses of 400 nm, 600 nm, and 800 nm) decreased by almost 1 MHz compared to the IDT-quartz case (39.909 MHz).

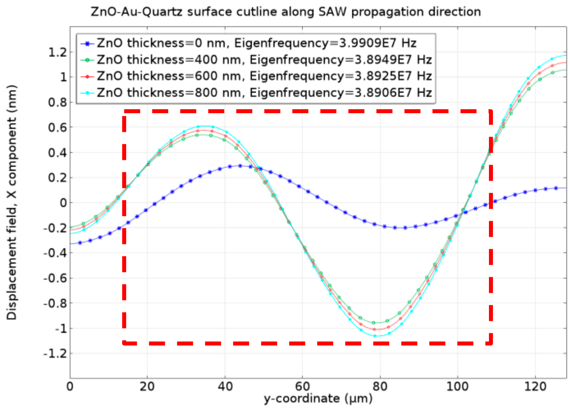

Figure S7-1. The comparison of three zinc oxide thicknesses for the X component displacement from the surface cutline along the y axis direction on the quartz surface. The amplitude exhibited a threefold increase along the propagation direction.

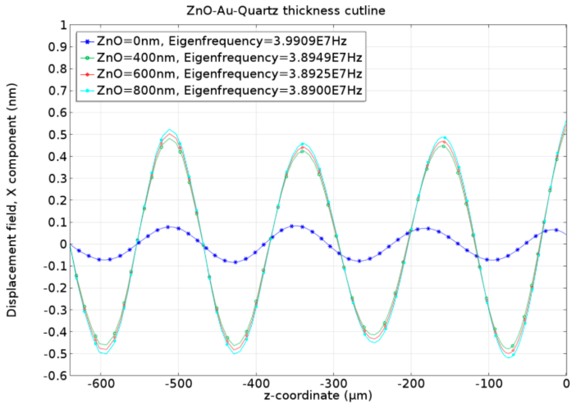

Figure S7-2. The comparison of three zinc oxide thicknesses for the X component displacement from the thickness cutline along the z axis direction. The amplitude exhibited a five-fold

increase along the thickness direction.

S8. A thin wall delay line model was built to analyze the displacement field response in the frequency domain. The x direction depth decreased from 256 μm to 16 μm. The split-electrode delay-line geometry on ST-90°X quartz substrate is 1024 μm wide (16 μm electrode width, 128 μm wavelength) in the y axis direction (SH wave propagating direction) with two sets of split-electrode IDTs at both ends (128 μm * 2 for both input and output IDTs) and four wavelengths metal-free propagation path at the center, 16 μm deep (the depth of each electrode strips) in the x axis direction, 750 μm tall in the z axis direction. A mesh size convergence test was also performed.

This delay-line model was focused on the frequency domain to reveal the total displacement (the absolute value from the three X, Y, and Z components) field response. The extracting data point location was at the center point of an uncharged and unbiased paired split-electrode from the output IDT set on the top surface of the ST-90°X quartz substrate. Figure S8-1 illustrates the total displacement field response for the delay-line model with 300 nm Au IDT on a 750 μm ST-90°X quartz, where the largest displacement response occurred at 39.438 MHz. The 5048 m/s wave speed was obtained from the frequency times 128 μm wavelength. This value was close to the value in Ref [27,28]**.** Figure S8-2 illustrates the case of 400 nm ZnO on the IDT/quartz, where the largest displacement response occurred at 39.174 MHz (wave speed: 5014 m/s). Figure S8-3 illustrates the case 1.5 μm Parylene C on the ZnO/IDT/quartz, where the largest displacement response occurred at 39.039 MHz (wave speed: 4997 m/s). The neighboring peaks around the resonant frequency for the ZnO/IDT/quartz case were more pronounced than the IDT/quartz case. Those peaks might be the side lobe energy leaking from the main resonant frequency.

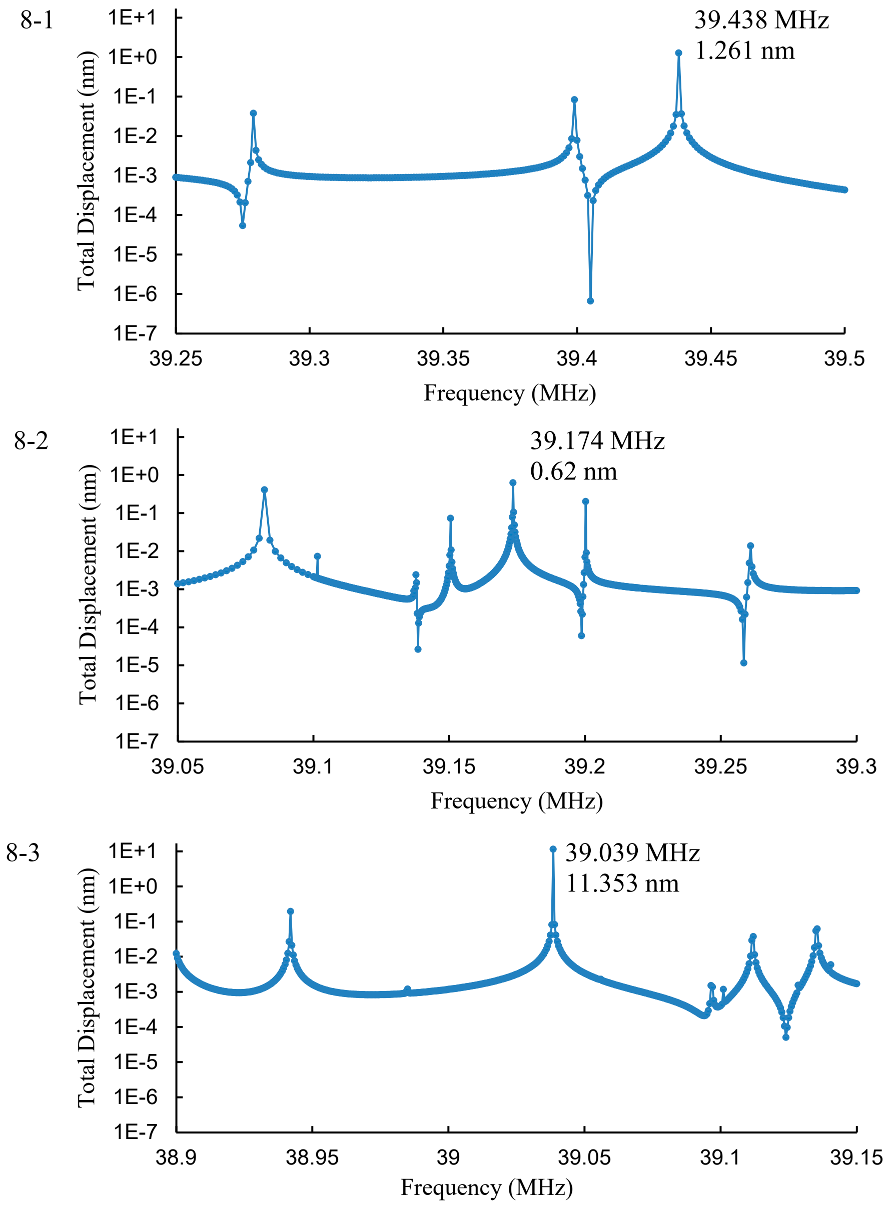

Figure S8. The total displacement field response for the delay-line model: 7-1. IDT/quartz. 7-2. ZnO/IDT/quartz. 7-3. Parylene C/ZnO/IDT/quartz.
